# A Network approach to find poor orthostatic tolerance by simple tilt maneuvers

**DOI:** 10.3389/fnetp.2023.1125023

**Published:** 2023-02-06

**Authors:** John M. Karemaker

**Affiliations:** Department of Medical Biology, Section Systems Physiology, Amsterdam University Medical Centers, Amsterdam, Netherlands

**Keywords:** multivariate analysis, radar plot, blood pressure, cerebral blood flow velocity, endtidal pCO2, tilt table, spaceflight

## Abstract

The approach introduced by Network Physiology intends to find and quantify connectedness between close- and far related aspects of a person’s Physiome. In this study I applied a Network-inspired analysis to a set of measurement data that had been assembled to detect prospective orthostatic intolerant subjects among people who were destined to go into Space for a two weeks mission. The advantage of this approach being that it is essentially model-free: no complex physiological model is required to interpret the data. This type of analysis is essentially applicable to many datasets where individuals must be found that “stand out from the crowd”. The dataset consists of physiological variables measured in 22 participants (4f/18 m; 12 prospective astronauts/cosmonauts, 10 healthy controls), in supine, + 30° and + 70° upright tilted positions. Steady state values of finger blood pressure and derived thereof: mean arterial pressure, heart rate, stroke volume, cardiac output, systemic vascular resistance; middle cerebral artery blood flow velocity and end-tidal pCO2 in tilted position were (%)-normalized for each participant to the supine position. This yielded averaged responses for each variable, with statistical spread. All variables i.e., the “average person’s response” and a set of %-values defining each participant are presented as radar plots to make each ensemble transparent. Multivariate analysis for all values resulted in obvious dependencies and some unexpected ones. Most interesting is how individual participants maintained their blood pressure and brain blood flow. In fact, 13/22 participants had all normalized Δ-values (i.e., the deviation from the group average, normalized for the standard deviation), both for +30° and +70°, within the 95% range. The remaining group demonstrated miscellaneous response patterns, with one or more larger Δ-values, however of no consequence for orthostasis. The values from one prospective cosmonaut stood out as suspect. However, early morning standing blood pressure within 12 h after return to Earth (without volume repletion) demonstrated no syncope. This study demonstrates an integrative way to model-free assess a large dataset, applying multivariate analysis and common sense derived from textbook physiology.

## Introduction

We make models of the world around and within us to understand what we observe and to predict future events. Network Physiology is no exception to that, as it is attempting to find (strength of) connections between various subsystems defining the functioning of an individual (Ivanov, 2021). Biological systems demonstrate extensive interconnectedness; in the end, almost any possible node or center of activity is connected to every other node. In network terms the heart may be considered a center node where the branching vessels are edges, connecting the center to the organs as dependent nodes. However, the vessels themselves are not passive conductors; they are adaptive by way of the autonomic nervous system, circulating vasoactive molecules, locally released peptides and stretch activation of smooth muscle. Moreover, they are innervated by pressure- (wall stretch-) sensitive nerves, giving them an active role as well (Malliani and Pagani, 1976; Malliani et al., 1983; Karemaker, 2017). This enumeration is just scratching the surface of the circulatory system. How to put this into an all-embracing model has been the objective of many studies, importantly started in the Annual Review of Physiology of 1972 where Arthur Guyton and others, literally unfolded an integrative model of the circulation on one 12 fold-out pages spanning print (Guyton et al., 1972). They described the various subparts of that model by differential equations, the whole to be programmed on a sufficiently large analog or hybrid computer to make it manageable for the computing capacity of the time. The coefficients of the differential equations are set: they are the **parameters** of the model, the resulting numbers (think “momentaneous blood pressure” for instance) are **variables** in the model, they can vary with time and changes in the parameters.

Description of a particular subject’s cardiovascular condition in terms of such a model, might be by quantification of as many biological variables under various stresses (exercise, orthostasis) as can be measured. Next, the model parameters should be tweaked to yield exactly those values under those circumstances for this particular subject. Ideally, the found solution (-s) should also hold under circumstances that had not been in the primary test set. Exactly this had been the idea when my research team and I measured a set of cardiovascular variables under various gravitational stresses, preflight in a group of astronauts, to predict their individual capacity to cope with the conditions of return to Earth after an about two-week stay in space. As is well-known, after return from even a short period of microgravity, orthostatic intolerance does occur to many astronauts (Buckey et al., 1996) to the point of them not being able to stand for 10 min without (signs of) presyncope or fainting. Our set of baseline cardiovascular data had been collected in 22 healthy participants, of whom 12 astronauts/-cosmonauts (Gisolf et al., 2005; Stok et al., 2019).

In the present paper a *network view* as alternative approach will be applied to find the ‘odd-man out’, without an elaborate model, but by comparing the dataset of each individual to the average set of all measured participants. To do this, a set of steady state variables (3–5 min averages) is chosen that can be considered to describe a person’s cardiovascular condition under gravitational stress, specifically values in supine, + 30° and + 70° head up tilted positions of mean arterial pressure (MAP), heart rate (HR), stroke volume (SV), cardiac output (CO), total peripheral resistance (TPR), cerebral blood flow velocity (FV) and End Tidal pCO_2_ (ETCO2). This constitutes a set of basic variables, which can be measured easily and non-invasively. Under-performance on those points during very limited gravitational stress can be considered a warning sign for what may happen under more extreme circumstances.

In the end, the prediction should be tested against reality: were the astronauts in the test population yes or no able to stand for 10 min immediately after return to Earth? Not to raise the expectations too high: the ones that returned safely to Earth (5) after around 2 weeks in Space were all able to do this, albeit sometimes with apparent difficulty (Gisolf et al., 2005). However, due to the Columbia disaster (2003), 7 participating astronauts did not survive re-entry, their return ended in catastrophe.

## Methods

### Participants

The group who participated in this study has been described extensively elsewhere (Gisolf et al., 2005; Stok et al., 2019). In short, 22 healthy persons (4f/18 m) took part after informed consent (ages 40 ± 8 years, height 177 ± 9 cm, and weight 72 ± 11 kg). Ethical approval had been obtained from the appropriate Review Boards. Of the participants 7 were NASA astronauts and 5 ESA cosmonauts, 10 healthy volunteers participated as controls.

### Procedures and physiological data

The procedures, measurements and source data handling have been described in the same earlier publications. For the present study of orthostatic tolerance 2 maneuvers from the whole set were chosen: passive head up tilt from supine to 30° and one to 70°. The former gives a load of 0.5 G, the latter of almost 1 G, while still allowing relaxed standing, fully supported by the tilt table. A stable period of 3–5 min was chosen and averaged, allowing about 2 min stabilization.

The following data were extracted from the database: heart rate (HR) and mean arterial pressure, MAP (both by Finometer™, TNO, BMI, Amsterdam, NL). From the pulse wave derived by pulse contour analysis, (Wesseling et al., 1993) were Stroke Volume (SV), Cardiac Output (CO) and total peripheral resistance (TPR), also known as systemic vascular resistance. Since the hand where blood pressure was measured, was kept in a sling at heart level, the hydrostatic difference between heart and the position of the Doppler probe attached to the head had been measured and taken into account to compute MAPbr, i.e. MAP at brain level, further specified as MAPbr30, or MAPbr70, for the + 30° and + 70° tilted position, respectively. Averaged cerebral blood flow velocity in the middle cerebral artery FV (=CBFV) was measured by transcranial Doppler (DWL, Germany) and end-tidal pCO2 from a continuously sampling capnometer at the nose ETCO2 (HP 1436A). The envelope of the maximum Doppler shift is output by the device as pulsatile flow velocity signal. This signal was beat-averaged and then time-averaged to the FV-number; End Tidal pCO2 was sampled from the continuous signal and also time averaged. This resulted in 3 sets of 7 variables per subject and per G-load of 0°, 30° and 70°.

### Calculations and representations

In common network representations, nodes are often presented as located on the outer rim of a circle, edges basically exist between each node and other nodes, filling the circle interior, e.g. (Rizzo et al., 2020). The same can be represented as a matrix where the nodes are the labels for the rows and columns. The matrix elements (numbers) represent the strength of the interactions. As in every network problem, the issue is to find the numbers that fill the matrix. Hereto, the following procedure was adopted: For all participants the chosen variables were entered into a [14, 22] matrix, 22 subjects, 2 × 7 physiological variables, normalized as % of the supine values. For statistical comparisons, %-normalized values were expressed as their Δ-value i.e., the deviation from the group average divided by the appropriate standard deviation. This resulted in one normalized [14 × 22] matrix (variables x participants), or, where appropriate, 2 matrices [7 × 22], considering the 2 levels of gravitational load separately. These matrices were entered into MS-Excel™ that was used for all computations, in particular the Pearson correlation coefficients to fill the network matrices and for the representation as radar-plots. Normality of the distributions of response values for MAP etc. was tested using the Shapiro-Wilk test (online available at https://www.statskingdom.com/shapiro-wilk-test-calculator.html).

To combine the variables per subject into one organized view, the *radar plot* was adopted. Here, the variables to be displayed are marked at the border of a circle; the radii connecting those to the center are spokes, used as *Y*-axis for that particular variable. Lines connecting the various values are there just to help the eye.

## Results

The results chapter will be divided into three sections: 1. A description of individual response patterns as apparent through the 7 chosen variables in 3 positions. 2. Analysis of the response patterns or coping mechanisms as a whole by a network approach. 3. Consequences of the network approach for the individual assessments.

1. Response patterns

**FIGURE 1:**
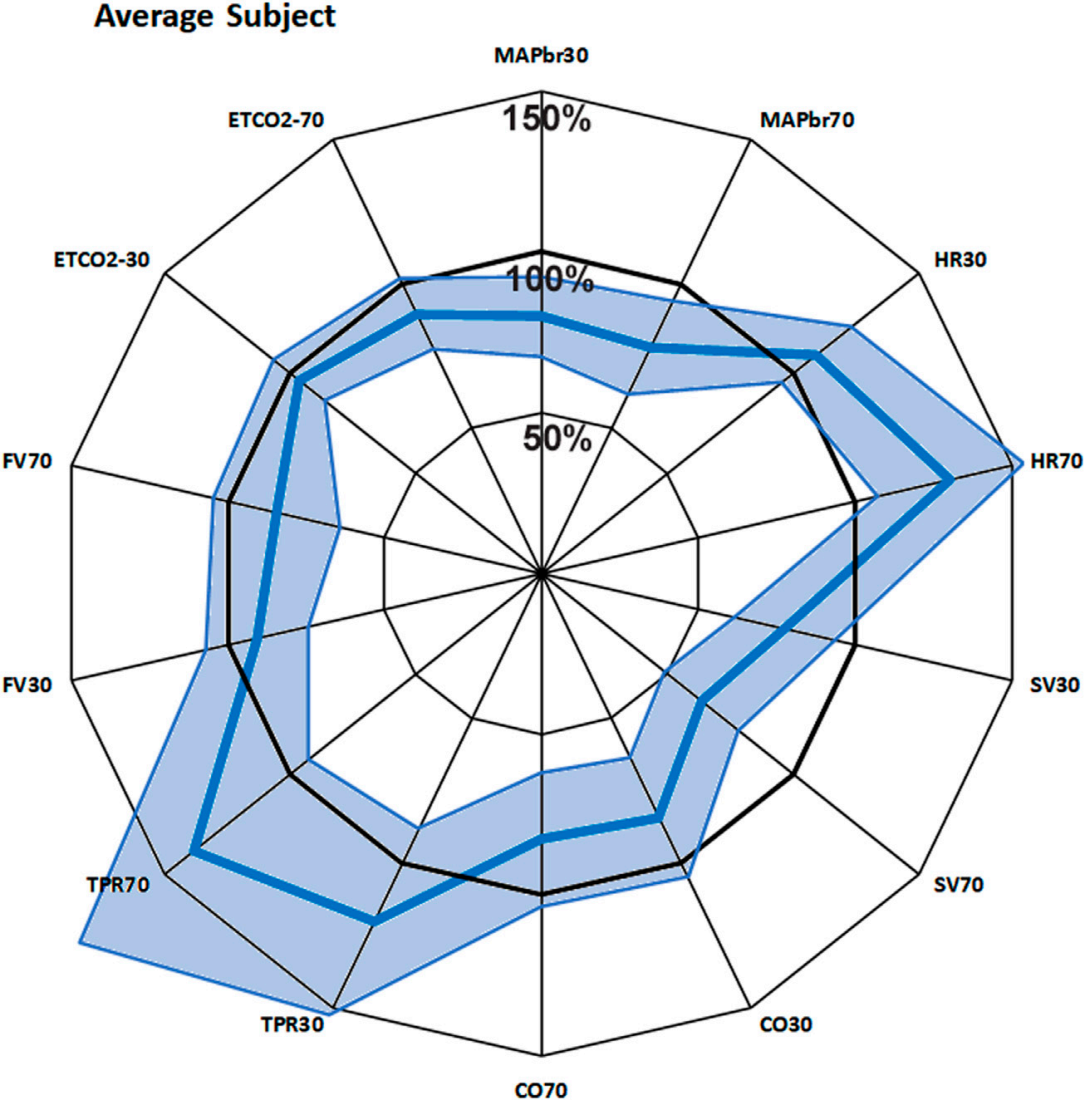
Bold blue line: average response of 22 subjects to + 30° and + 70° head up tilt, juxtaposed for each measured variable. The spokes (radii) represent the axes, from 0% to 150% of supine value. The line joins the various points on the axes. The light blue area shows the 95% confidence interval. MAPbr30, MAPbr70: mean arterial pressure at brain level, % remaining pressure of supine. Same idea for HR (heart rate), SV (stroke volume), CO (cardiac output), TPR (total peripheral resistance), FV (cerebral blood flow velocity) and ETCO2 (End tidal pCO2).

To get an immediate overview of the group response to the 2 interventions (+ 30° and + 70° head up tilt), the averages, expressed as % of the supine values, are presented as the fat blue line in a radar plot as Figure 1. The values for the same variable at + 30° and + 70° tilt are put side by side. This response will be referred to as “Mr. Average” in the next paragraphs.

The 100-circle marks the “no-change” level, the axis is from 0% to 150% of supine values. Each value has its own axis as a radius of the circle, e.g., MAPbr30 shows MAP dropping to 80%, MAPbr70 drops more to 78% of supine. However, it must be noted that MAPbr will drop even if MAP at heart level remains constant - it does not, normally it slightly increases (ten Harkel et al., 1993); the apparent drop is due to the hydrostatic effect of the column of blood between heart and brain level which was taken into account for comparison with the cerebral blood flow velocity values (FV). Even though SV is seen to drop to 77% and then to 64%, this is compensated by HR, maintaining CO almost equal. TPR is increased, FV and ETCO2 are decreased. The numbers, including the original supine values are presented in Table 1. The light blue areas in Figure 1 indicate the ± 2.074 x standard deviation, assuming normal distributions (only HR30 failed that test, due to one high and one low outlier; therefore, the s.d. was used anyway). The broad range around the averages might give the wrong impression: no participant had all values near the lower or higher border. Probably that would not represent a ‘physiological’ response to a gravitational challenge. It merely indicates that individual coping mechanisms can be widely different and still lead to a stable situation under the imposed G-stresses.

**TABLE 1:** Average results over all subjects for the various variables (column 1). Column 2 has the actual supine values, with standard deviations (column 3). Columns 4 and 6 show the % of these supine values in + 30° and + 70° head up tilted position, respectively.

	*Supine*	*s.d*	*+30° tilt (%)*	*s.d*	*+70° tilt (%)*	*s.d*
MAP	77.1 mmHg	7.15	79.9	5.94	78.0	7.65
HR	61.9 bpm	12.36	109.0	6.66	130.4	11.13
SV	69.0 mL/beat	8.37	77.3	7.74	63.6	7.01
CO	4.3 L/min	1.02	84.2	9.92	82.7	10.06
TPR	1.1 med. units	0.22	120.1	15.40	138.3	21.92
FV	61.2 cm/s	15.53	90.6	7.85	84.6	9.68
ETCO_2_	37.9 mmHg	3.02	96.3	4.85	89.5	5.83

To find these differences in response patterns and possible outliers, Δ-values were calculated: the appropriate %-average was subtracted from each %-value, then normalized by division by the standard deviation, resulting in Δ-values. The squares of all Δ-values per subject were added up and the group was ranked according to this total. In mathematical terms, the square root of these numbers gives insight into the distance of each individuals’ values vector to the average.

**FIGURE 2:**
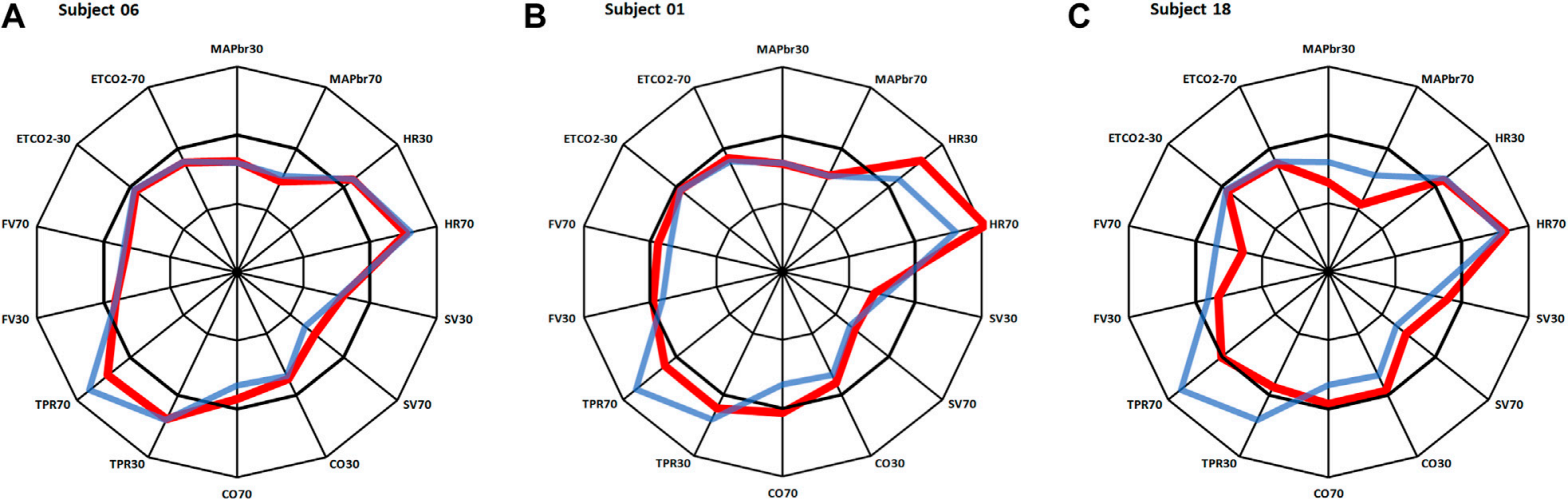
Same layout as in Figure 1. Red lines: results for 3 individual participants, in blue: average results as in Figure 1. **(A)**: almost same response as Mr. Average; **(B)**: deviations in heart rate; **(C)**: deviations in MAP, TPR and FV. More details on these participants in the text.

In Figure 2 three response patterns thus computed are shown for 3 individual participants. Figure 2A shows an almost “Mr. Average”, all Δ-values but one (SV70) are within 1 times standard deviation. The results in Figures 2B, C require some closer inspection. In particular the heart rate response in Figure 2B might be indicative of POTS (postural tachycardia syndrome), however the well-maintained MAPbr, CO and FV tell differently. A check of this participants original data gave a good explanation for this outlier in HR: it went up from supine 62.4 to 81.4 bpm at 30° and then to 95.5 at 70°, demonstrating withdrawal of cardiac vagal activity rather than sympathetic overactivity to heart and vessels, as further demonstrated by the reaction of TPR, which is a purely sympathetic effect (Karemaker, 2017). However, the pattern observed in Figure 2C raises some real concern: both MAPbr30 and MAPbr70 show Δ-values around −3 times the standard deviation, TPR and FV are low for both tilt angles well. More about this participant below.

2. Multivariate analysis; how does the Network counteract gravity?

The idea of network analysis is to find connections within a given system and their strength, depending on the conditions. To counteract gravity, a series of adaptations is called into play, as evidenced in the previous paragraphs. The dataset used here has been assembled to measure a set of (non-invasive) variables that, together, is involved in orthostatic tolerance. The questions to be answered now are: which variables are the most important players in this concert and how do they play together?

The input data are 2 [7,22] matrices, 22 participants, 7 variables measured in 2 circumstances (+ 30° and + 70° tilt up). The one-on-one correlation results for the 2 matrices are shown in Table 2. Since such a correlation matrix is symmetrical by definition, only half of it is filled, the other values can be mirrored in. For n = 22 a correlation value of 0.360 is significant at *p* = 0.05, a correction for multiple comparisons should be applied; however, the exact correlation is here, strictly speaking, of no consequence. The values around 0.36 and higher are printed in bold, just to stress the strength of their coherence in this group and this experiment.

**TABLE 2:** Correlation matrices for the results in the + 30° (2A) and +70° (2B) position respectively. Numbers show correlations between 7 variables as indicated for 22 participants. e.g., correlation between (row) CO70 and (column) HR70 is 0.512. The inversion: column CO70 vs. row HR70 would have the same result and is left open. Bold printed numbers indicate statistical significantly correlations (but see text).

**TABLE 2A**	*MAPbr30*	*HR30*	*SV30*	*CO30*	*TPR30*	*FV30*	*ETCO*_*2*_ *-30*
MAPbr30	1.000						
HR30	−0.174	1.000					
SV30	0.009	−0.003	1.000				
CO30	−0.092	**0.498**	**0.864**	1.000			
TPR30	**0.378**	**−0.455**	**−0.788**	**−0.914**	1.000		
FV30	**0.404**	−0.232	−0.248	**−0.356**	**0.468**	1.000	
ETCO_2_-30	0.028	−0.344	−0.069	−0.241	0.215	**0.562**	1.000

**TABLE 2B**	*MAPbr70*	*HR70*	*SV70*	*CO70*	*TPR70*	*FV70*	*ETCO*_*2*_ *-70*
MAPbr70	1.000						
HR70	−0.246	1.000					
SV70	**−0.426**	−0.248	1.000				
CO70	**−0.555**	**0.512**	**0.704**	1.000			
TPR70	**0.660**	−0.336	**−0.767**	**−0.927**	1.000		
FV70	0.317	**−0.360**	0.281	−0.022	0.093	1.000	
ETCO_2_-70	−0.102	−0.311	**0.577**	0.273	**−0.360**	**0.545**	1.000

Within the complex MAP-HR-SV-CO-TPR there are many obvious correlations, since all numbers are derived from the formula MAP = CO*TPR where CO = HR*SV. The ones that have actually been measured are HR and MAP; SV is derived by pulse contour analysis, the rest (CO, TPR) derives from there. For the circulation as a whole, MAP is the most important variable to be maintained, as long as the other three (HR, CO, TPR) remain within physiological limits. Remarkably, the correlations between CO and HR&SV are lower for HR than for SV, demonstrating more individual variability in the HR-response than in the SV-response.

**FIGURE 3:**
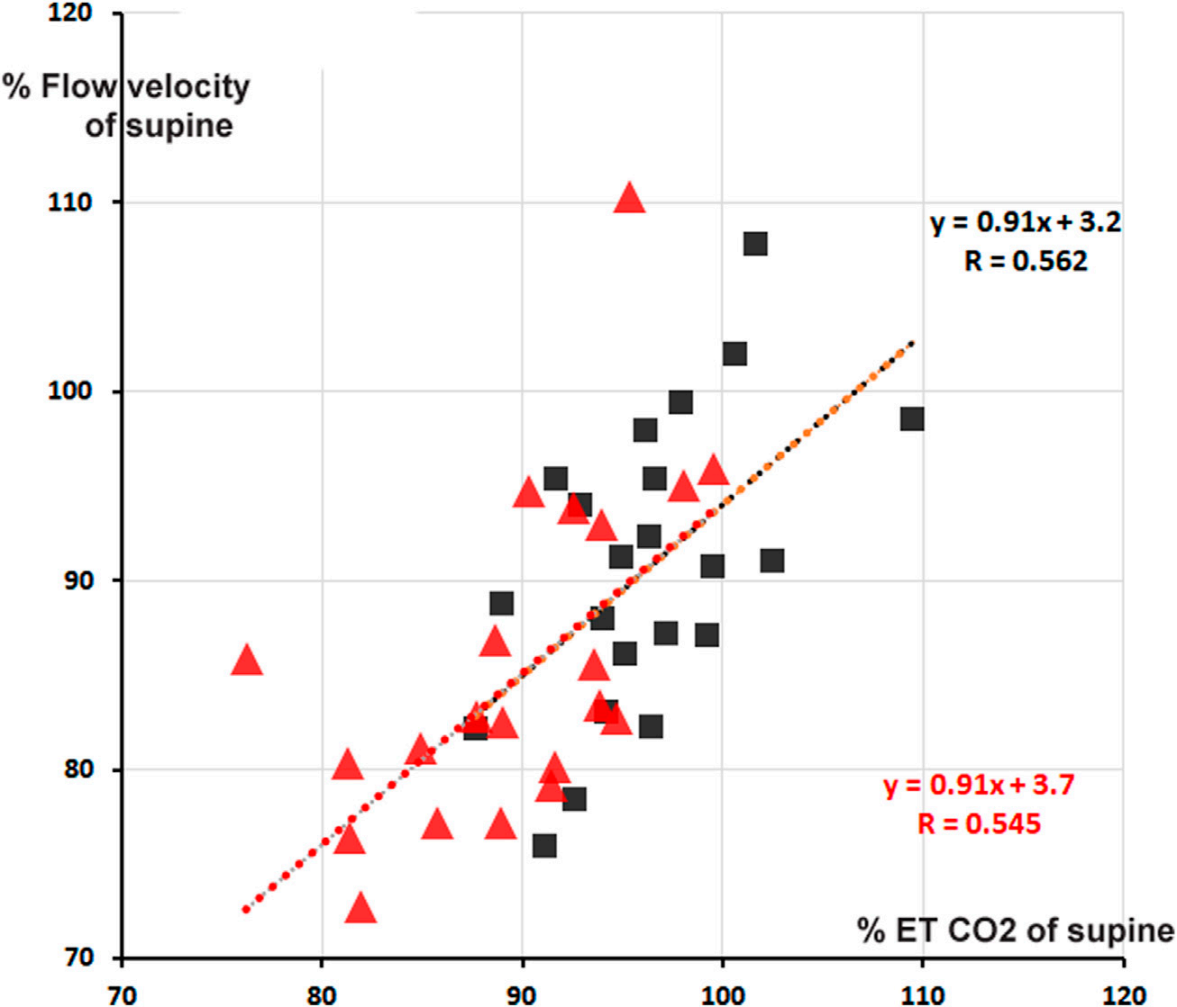
Percentage cerebral blood flow velocity (FV) from supine as function of % end tidal pCO2 (ETCO2). Red triangles: + 70° tilt, black squares + 30°. In red and black are the respective computed regression lines with correlation coefficients (*cf.* Table 2). The two regression lines almost coincide, apart from a small shift.

Cerebral blood flow velocity (FV) is positively correlated to MAPbr, however, only significant in the + 30° tilt position; (the change in) ETCO2 is an even stronger determinant, one that also holds in the + 70° position. Brain vessels are well-known to be sensitive to changes in blood-CO2 levels, as shown in hyperventilation which may lead to syncope (Levine et al., 1994). The amount of %-FV drop per %-ETCO_2_ drop is accordance with existing literature (Figure 3), (Lodi et al., 1998).

When judging the various correlations in Table 2, one may wonder about the topology of the network: there is a cluster of those that have most of the significant correlations with other variables (i.e., TPR, CO, MAP) where MAP may be considered the most tightly regulated variable and CO&TPR are the ones that keep it under control with some more room for individual patterning. TPR is mainly sympathetically determined, CO by SV and HR. Of these two SV is bound by venous return to the heart and the given cardiac structure, HR is dependent on the interplay between sympathetic and parasympathetic activity.

FV is a story of its own: obviously dependent on MAP as driving pressure, and furthermore on end tidal pCO_2_. The correlations between FV and other variables are inconsistent, a striking negative correlation between FV70 and HR70 (−0.360), less so for FV30 and HR30 (−0.232). The expected correlation with CO (Ogoh et al., 2005) was not observed. In topological terms one may conclude that FV is a distant loner, separate from the central cluster around MAP and CO.

3. Individual response patterns

**FIGURE 4:**
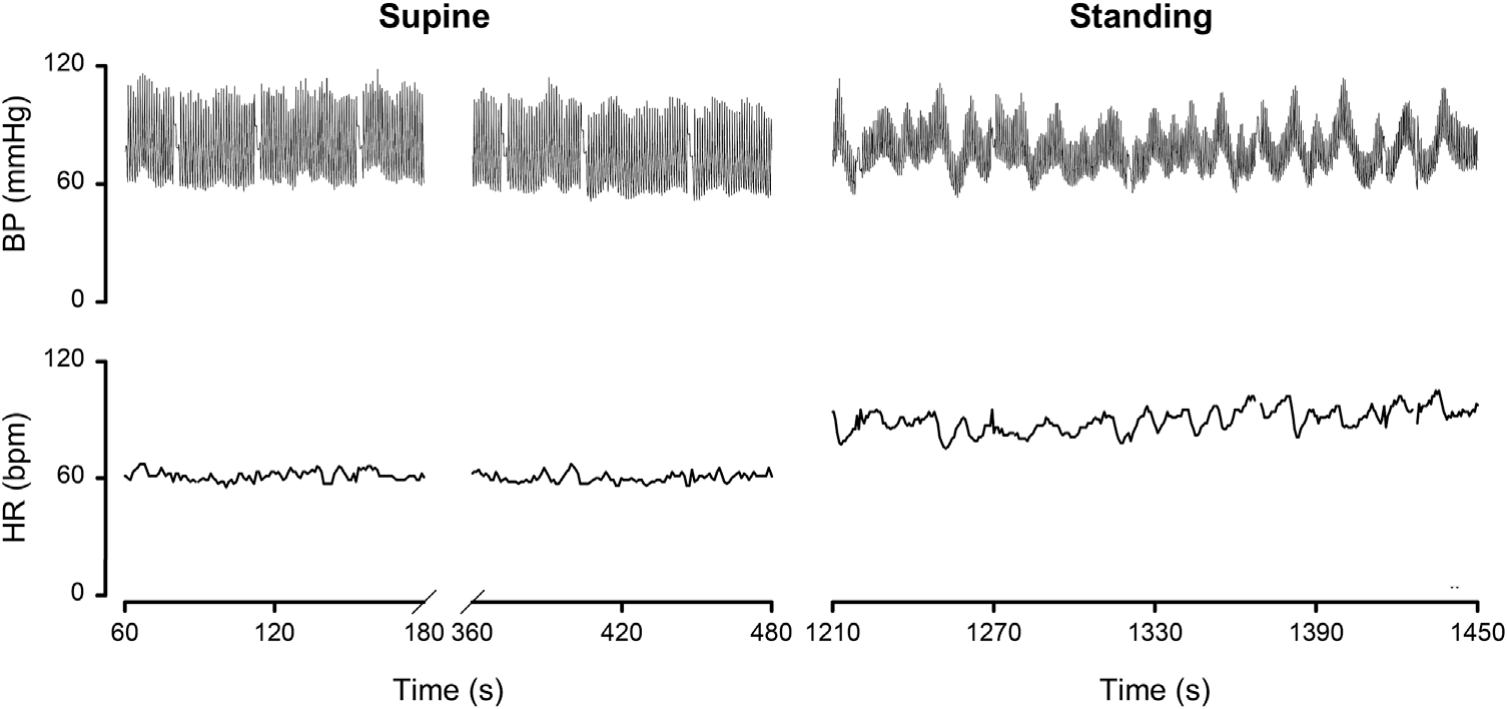
Finger blood pressure (BP) and heart rate (HR) by Portapres™ in subject 18, cosmonaut, on the first morning after return from a 10 days mission to the ISS. Details in the text. Figure adapted from the one in (Gisolf et al., 2005). Reproduced by permission.

By dividing the % values minus the averages by their respective standard deviations (Table 1), the normalized deviation from the mean **Δ**, is found, making the numbers better tractable for statistical analysis. Now one may ask questions like: how many participants had all Δ values between ± 2.074 (Student-t two-tailed for *p* < 0.05)? The answer is 13. Next step is to analyze the remaining 9 and judge the implications of the high or low Δ’s. Participant nr.01 is an example here, as demonstrated in Figure 2B. To understand the deviating HR-responses, the original numbers were looked up to exclude postural tachycardia. Checking the whole [14,22] matrix in this fashion, only participant nr.18 (cf; Figure 2C) remained as suspect for orthostatic problems. In retrospect we can clear him of all charges: he is one of the five ESA-cosmonauts who participated in this experiment. We were allowed to measure his (finger) blood pressure and heart rate response at the first standing up in the morning after arrival back from an about two weeks mission to the International Space Station. Between landing and going to sleep no volume repletion had taken place. Blood pressure was variable but well maintained and, even after 10 min of standing, heart rate did not exceed 115 bpm (Figure 4). The reverse, i.e., no alarm at the preflight recordings (or in the present, *post hoc* analysis), but still orthostatic problems after return to Earth, did not occur either, even though all returning cosmonauts had variable blood pressure with small pulse pressures (stroke volumes) and high heart rates (Gisolf et al., 2005). Comparison of the astronaut/cosmonaut group to the healthy control group revealed no significant differences.

## Discussion

The cardiovascular system is a network of networks: heart and vessels, respiratory system, autonomic nervous and local (tissue-) metabolic control. It can only serve its many functions properly, when all these systems work in concert, to meet the demands of ever-changing circumstances of daily life. To the physiologist who is researching (parts of) the orchestra and to the medical doctor who is called in when adaptation fails, this interconnectedness poses a challenge. In this study stability of orthostatic tolerance was tested, to find subjects “at risk” under future extreme circumstances (re-adaptation to gravity) that have not been tested as such. Therefore, the “odd man out” approach was used, to find those subjects in a group of healthy participants who had extreme reactions to orthostatic tests.

In a recent review on orthostatic intolerance in astronauts (Jordan et al., 2022), the authors state that *“It appears that orthostatic intolerance following space flight is not explained by a single mechanism”*. I could not agree more. That is the reason why the present study tried to combine a set of basic cardiovascular variables. The same approach might be applied to all available measurements in the medical data bases of the various space agencies, given sufficient privacy protection and anonymization. This wealth of data has, to the best of my knowledge, not be ‘mined’ yet.

Usually, if a patient is to be tested for syncope due to orthostatic intolerance, a long-lasting tilt test will be performed. This may last until symptoms of presyncope (or actual syncope) up till 30 min; if that shows insufficient, a sublingual nitroglycerin containing spray is given to induce vasodilation (Gisolf et al., 2004). In the present case, where astronauts are involved, such rigid testing is not allowed in medical experiments. Therefore, we have here only the results of short-lasting tilt tests. Still, it allows us to study the process of adaptation to standing and compare that in a group of healthy participants. For this testing a set of variables was chosen that represent important aspects of the process. Arterial pressure and brain blood flow are, obviously, key parameters that need to be held within working range. Standing heart rate is known to be higher in patients at risk (ten Harkel et al., 1993), vascular resistance and end tidal pCO2 may get lower than normal early in the process. Since excessive blood pooling may be a factor as well, lowering of stroke volume is also a sign. These factors all have their place in the network of factors (autonomics, respiration) which together come into play during standing.

The new approach, suggested by Network Physiology to put complex biological problems into network descriptions (Ivanov, 2021), does not yet have a mathematical technique of its own. A number of proposals and examples have been published: for instance, when the particular properties of the dataset clearly required application of non-Normal statistics (West, 2014), Granger Causality estimation (Antonacci et al., 2021), time delay stability testing (Bashan et al., 2012) or a combination of Wavelet Phase Coherence and Conditional Mutual Information (Clemson et al., 2022), to name but a few of the applied methods. In the present study I tried a new way to treat an ensemble process while maintaining simplicity in presentation and computations. Since the values entered in the data matrix here are more or less steady state mean values, no variabilities or complex time interactions can be deduced. Nevertheless, some new and practical conclusions have been derived, even though the original goal to develop a network for the prediction of orthostatic intolerance after space flight has not been reached.

Radar plots have been used in medical literature (Saary, 2008) to represent multidimensional data. The technique allows application of different scales for the various radii (axes) that show the different properties. In the present study all parameters have the same dimension and scale, the order of the different parameters along the circle is, to a certain extent, arbitrary: shuffling will result in different shapes of the line plot, but it will not alter its meaning. The connecting lines are just there to “guide the eye”, they have no intrinsic significance. However, the present order was not chosen by chance: on top is MAP, the most important of cardiovascular variables, flanked by ETCO2, a respiratory variable, but sensitive to pulmonary filling and, consequently, cardiac diastolic filling (Gaffney et al., 1981; Grmec et al., 2009). Turning clockwise are HR, independently measured and SV, derived by pulse contour (Wesseling et al., 1993). The product of these two is CO. TPR is opposite to HR, a sympathetic marker *versus* a combined parasympathetic/sympathetic one.

Correlation matrices and their translation into various types of network plots have a history, in particular in analysis of psychometric data. The software to do this for very large datasets is readily available for various mathematical packages (e.g., R, Matlab) as qgraph (Epskamp et al., 2012). The present dataset is sufficiently small to do without this visualization; the correlation matrices themselves (Tables 2A, B) do not require beautiful, colored displays, at the risk of distracting from the underlying physiology.

The correlation matrices in Table 2 show interesting details. The high negative correlation between CO and TPR was to be expected: if MAP does not change too much from supine, the applied formula MAP = CO * TPR predicts an inverse relationship between the two (“Ohms law of the circulation”). HR is (marginally) negatively correlated to FV (−0.232 at 30° and −0.360 at 70°). One may speculate from this about the influence of the sympathetics on both: higher HR, higher sympathetic activity, lower FV? The only stable correlation for FV is ETCO2 (Figure 3), and even then, only some 30% of variation (*R* ^2^) is explained. That is still more than MAP vs. FV, explaining only 16% in 30° (R = 0.404) and 10% in 70° (R = 0.317). The other correlations are not equally strong in both positions or even change sign. This shows the advantages of the availability of measurements in two tilt angles, even with drawbacks like possible time-order effect, which was not considered in this experiment.

## Limitations

Relaxed tilt table testing, like all testing in more or less resting conditions, is a limitation of the proposed assessments here. This shows most clearly in the results of participant 18 (Figure 2C). Despite his poorly maintained blood pressure, CO was elevated, thanks to increased SV, but TPR was low. These factors combined are compatible with the condition of a slender, tall man, who is active in endurance sports: the increased vascular bed of the legs is now a disadvantage, since blood is not actively pumped out by movement, be it running or cycling etc. Increased SV (thanks to adaptive cardiac enlargement) may produce sufficient CO at moderate heart rates (Naylor et al., 2008; Whyte et al., 2008) to maintain blood pressure. But not so at rest on a tilt table.

This study is but a simple example with a modest group of only 22 healthy participants; it comprises only 4/22 women (2 astronauts and 2 controls). This choice was not up to our research group, but had been dictated by the selections made by the space agencies NASA and ESA.

## Conclusion

These experiments and the presentation of their results (for instance as radar plots) demonstrate how a network physiology approach, putting all available data in one, coherent schematic, may be of help in the interpretation of physiological findings. The correlation matrices reveal the internal structure of the set, helped by some basic physiological knowledge. MAP and ETCO2 in the tilted-up positions have the lowest standard deviations, they are controlled by the baroreflex and chemoreflex, respectively. The adaptation of other variables (HR/SV/CO/TPR) shows more variability between subjects, obviously pointing at individual coping patterns.

However, it must be stressed that correlations are not causations. The present experiments had no testable endpoint for all participating astronauts/cosmonauts, in part due to the tragic ending of the Columbia space mission in 2003. Data sets that do have such endpoints for all subjects lend themselves to a neural network analysis. In those cases, the correlation coefficients in the descriptive matrices may be replaced by those from the neural network. However, failing those endpoints, the current approach shows promise for other data sets, in larger and more diverse groups.

## Data Availability

The data analyzed in this study are subject to the following licenses/restrictions: The dataset as such contains identifiable data related to specific astronauts/cosmonauts. Depending on the intended use of (parts of) the data these should either be anonymized or data transfer should be restricted. Requests to access these datasets should be directed to JK, PhD (author), j.m.karemaker@amsterdamumc.nl.
